# Mixed convective flow of a magnetohydrodynamic Casson fluid through a permeable stretching sheet with first-order chemical reaction

**DOI:** 10.1371/journal.pone.0265238

**Published:** 2022-04-01

**Authors:** Anwar Saeed, Ebrahem A. Algehyne, Musaad S. Aldhabani, Abdullah Dawar, Poom Kumam, Wiyada Kumam

**Affiliations:** 1 Center of Excellence in Theoretical and Computational Science (TaCS-CoE), Faculty of Science, King Mongkut’s University of Technology Thonburi (KMUTT), Bang Mod, Thung Khru, Bangkok, Thailand; 2 Department of Mathematics, Faculty of Science, University of Tabuk, Tabuk, Saudi Arabia; 3 Nanotechnology Research Unit (NRU), University of Tabuk, Tabuk, Saudi Arabia; 4 Department of Mathematics, Abdul Wali Khan University, Mardan, Khyber Pakhtunkhwa, Pakistan; 5 Department of Medical Research, China Medical University Hospital, China Medical University, Taichung, Taiwan; 6 Applied Mathematics for Science and Engineering Research Unit (AMSERU), Program in Applied Statistics, Department of Mathematics and Computer Science, Faculty of Science and Technology, Rajamangala University of Technology Thanyaburi, Thanyaburi, Pathumthani, Thailand; Tongji University, CHINA

## Abstract

This research article presents the magnetohydrodynamic Casson fluid flow through an extending surface embedded in a porous medium. Furthermore, the Casson fluid flow is investigated under the effects of thermal radiation, Joule heating, viscous dissipation, and chemical reaction. The analytical solution of the modeled problem is utilized with the help of homotopy analysis method (HAM). The convergence region of the applied technique is portrayed graphically. The impacts of the embedded factors on the flow profiles are exhibited with the help of figures. Furthermore, numerical values of the surface drag force, heat, and mass transfer rates are highlighted via table. The results show that the augmented Darcy number, Casson and magnetic parameters have declined the velocity profile of the Casson fluid flow. Growth in Brownian motion augments the chaotic motion amongst the particles due to which the kinetic energy of the particles transforms to heat energy which consequently augmented the thermal profile, while reduced the concentration profile. The mass and energy profiles are positively effects with the increment of thermophoresis term. And the growing values of chemical reaction and Lewis number cause a reduction in the diffusivity of mass of fluid due to which less transfer of mass takes place that weakens the concentration layer thickness and declines the concentration profiles.

## 1. Introduction

During the past few decades, the mass and heat transmission past a permeable and extending sheet has achieved a significant response from numerous researchers for its important applications in industry and engineering technology. Carbonell and Whitaker [1] inspected the transmission of heat and mass for permeable medium with the main focus on the energy and concentration equations in their study. Yaglom and Kader [2] have investigated the transportation of mass and heat within a turbulent fluid flow and a rough wall by employing a high Reynolds number. It has been observed in this work that, more disturbances have been produced due to the roughness of the wall that has augmented the transmission of heat and mass in comparison to a smooth wall at similar values of Prandtl and Reynolds numbers. Bandaru et al. [3] have scrutinized the thermal and mass flow from a rotary cone to fluid transport through a permeable medium by using thermophoresis and nonlinear convective impacts. The thermal flow is more prominent and influential than mass transportation. Krishna et al. [4] have used a permeable vertical sheet to investigate the temperature and concentration for a second grade fluid. Ahmad et al. [5] observed the transportation of heat for hybrid nanoparticles flow upon a porous surface.

In fluid flows, the transportation characteristics have been described by two different models that are Buongiorno model [6] and Das, Tiwari model [7]. The main focus in the former model is upon the upsots of thermophoresis and Brownian motion regarding the flow of fluid under the influence of numerous flow conditions. Khan et al. [8–11] conducted remarkable work in the field of energy and mass transference for fluid flow employing different geometrical and several flow conditions views. It has been highlighted in these investigations that the energy field has augmented by varying the Brownian motion parameter and thermophoretic effects, which on the other hand has declined the mass flow rate. Mittal and Petal [12] have discussed the impacts of these two terms over mixed convection bi-dimensional MHD fluid flow by taking the heat generation and nonlinear radiations. It has been observed in this study that the concentration characteristics have declined while the thermal profile upsurge with augmented values of thermophoretic. Ashraf et al. [13] have inspected the effects of these two terms upon the flow of fluid using viscous dissipation in the closed vicinity of the stagnation point. Abdelmalek et al. [14] have used the electrically conducted visco-inelastic nanoparticles flow on a stretching surface under the influences of thermophoretic effects. It has been detected in this inspection that flow has reduced and thermal profiles have increased with augmentation in magnetic strength. Rashidi et al. [15] presented the comprehensive analysis of the thermophysical properties of hybrid nanofluids. Mahdi and Nazari [16] investigated the water-based Ag nanofluid flow by using an artificial neural network.

The combination of chemical reactions with the analysis of energy and mass allocation plays a momentous role in flow problems. For its importance and extensive applications, it attained the remarkable attention of researchers and scientists in the last few decades. These applications include different engineering progressions such as cooling of electrical equipment and devices, transpiration of humans, chemically catalytic reactors and aircraft propulsion devices, etc. Gangadhar and Bhaskar [17] have presented a two-dimensional mathematical model to discuss the thermal and mass flow for MHD fluid flow upon a porous and expanded surface. It has been revealed in this work that, the flow profile has declined sharply due to growth in the magnetic field while the thermal flow has amplified in this process. Seth et al. [18] inspected the upshot of chemical reactions upon thermal and mass transmission for convective fluid flow past a moving porous sheet. In this work, a closed form solution has been obtained for flow, energy, and concentration equations by using Laplace transformation. Moreover, the descriptions for Sherwood and Nusselt numbers have also been determined in this work. Reddy et al. [19] have presented the profiles of thermal and mass transmission for MHD fluid flow by using chemical reactions on a rotating permeable disk. The thermal radiation upshot and partial slip has also been incorporated in this work and has established that flow has reduced while thermal flow has improved with the increment of solid nanoparticles. Raju [20] has inspected the time dependent MHD fluid flow on a penetrable and inclined sheet by considering the chemical reaction upshots in the concentration equation. In this study, the Darcian flow model for permeable surfaces has also been used in the flow system. Punith Gowda et al. [21] surveyed the fluid flow and heat communication for non-Newtonian fluid using a chemical reaction. The results of this work revealed that flow has augmented and thermal profile has declined with escalating values of Marangoni number. Moreover, it has been perceived that the flow profiles condensed with the expansion in permeability term. The readers can further study the effects and benefits of chemical reactions upon fluid flow system in Refs [22–26].

A surface that contains small void spaces (pores) is characterized as a permeable surface. The idea of the porous medium is extensively used in applied sciences such as filtration purposes, petroleum engineering, petroleum geology, geophysics, biophysics, and biology, etc. Due to its importance in the field of applied sciences, many investigations have been carried out with the core focal point on thermal flow and transference of mass in fluid motion upon the porous medium. Bejan and Khair [27] have discussed the heat transition for the fluid flow over a porous texture. Chaudhary and Jain [28] have investigated the fluid flow past a plate implanted in a permeable region. It has been established in this investigation that magnetic effects have supported the thermal characteristics and have opposed the flow of fluid. Jiang et al. [29] reviewed the MHD unsteady flow of fluid inside a penetrable surface with the influence of Hall current upon thermal flow. The concept of fractional derivative along with constitutive equations has been employed in this work. Kumar et al. [30] have inspected the cross flow for MHD fluid upon an accelerating upright surface in an absorbent surface using Hall current. It has been observed in this work that mass transfer of fluid has been maintained by enlarging credit of Soret number and has been opposed with augmentation in Dufour number. A comparative study has also been carried out in this investigation with a fine agreement with published results. Haq et al. [31] scrutinized the convective thermal flow of ferrofluid across a porous exterior. In this study, the thermal radiation has been incorporated in the temperature equation that has supported the flow and temperature profile.

After a careful review of the above literature, we are in a position to present the steady and incompressible two-dimensional flow of magnetohydrodynamic Casson fluid through an extending sheet inserted in an absorbent medium. The fluid flow has exposed with the impacts of Brownian motion and thermophoresis by using the idea of Buongiorno’s model. The influence of chemical reaction has also been incorporated mathematically in the concentration equation. At the end of this analysis, we will respond to the subsequent research queries.

1. Does the Casson parameter strengthen the momentum boundary layer?
2. How does the Casson fluid flow behave against the magnetic field using porous media?
3. How does the Casson fluid flow behave against the thermophoretic and Brownian motion parameters?
4. What is the effect of Darcy number on the thermal profile of the Casson fluid flow?
5. What is the impact of chemical reaction constraint on the mass profile of the Casson fluid flow?

## 2. Problem formulation

**Fig 1:**
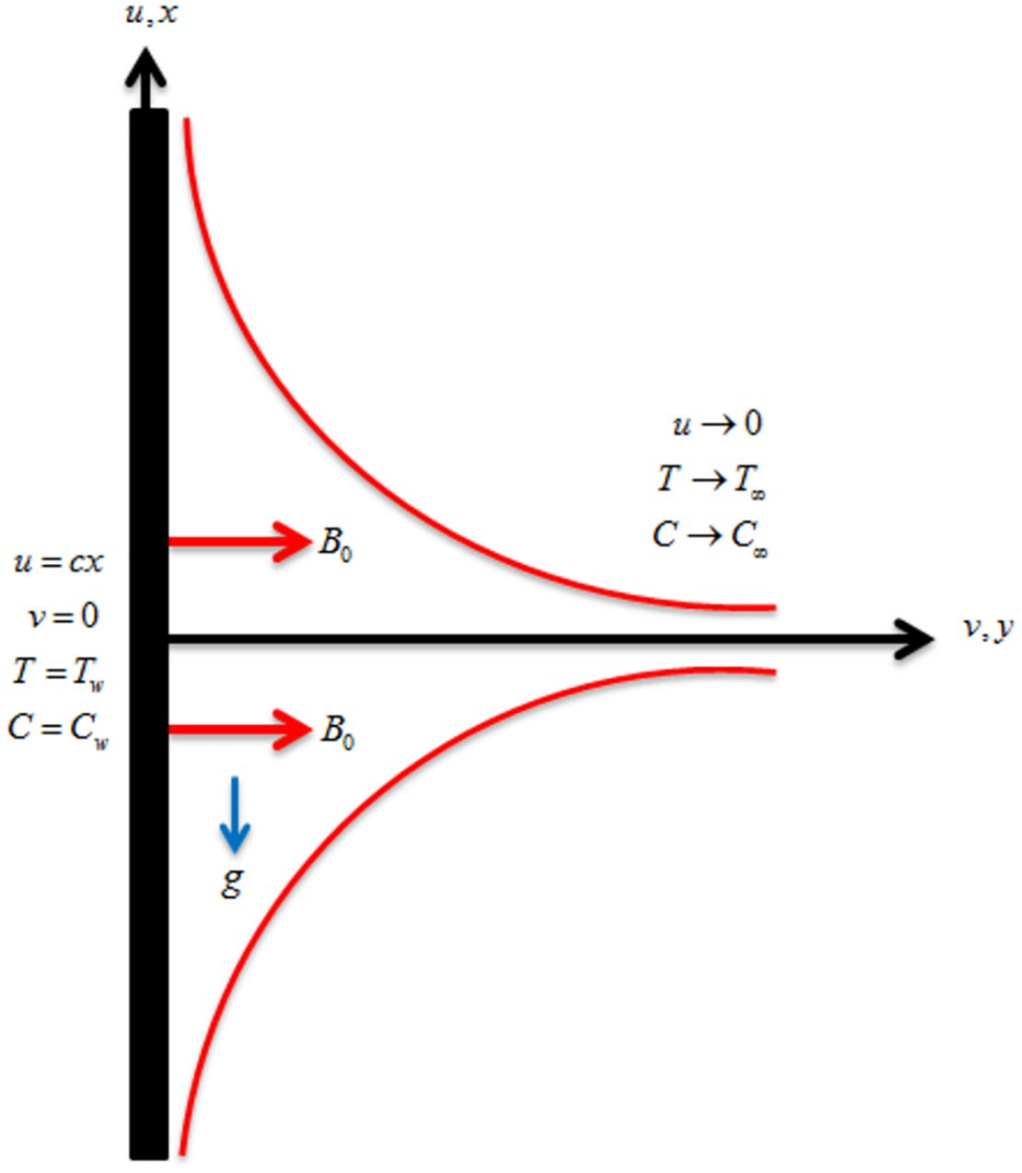
Geometry of the fluid flow.

Consider the steady and incompressible magnetohydrodynamic and thermally radiative flow of Casson fluid across a stretching sheet embedded in a permeable medium. The extending velocity of the sheet is defined as *u*_*w*_ = *ax* where *a* is the positive constant. In the *xy*–plane, *x* is taken along the sheet and *y* is taken perpendicular to the stretching sheet as shown in Fig 1. A magnetic field of strength *B*_0_ is applied normal to the fluid flow. The gravitational force *g* acts in downward direction. The surface temperature is represented by *T*_*w*_ whereas the ambient temperature is signified by *T*_∞_. Also, the surface and ambient concentrations are respectively represented by *C*_*w*_ and *C*_∞_. The leading equations are defined as [32, 33]:

$$\frac{\partial u}{\partial x}+\frac{\partial v}{\partial y}=0,$$ (1)

$$u\frac{\partial u}{\partial x}+v\frac{\partial u}{\partial y}=\frac{\mu_{f}}{\rho_{f}}\left(1+\frac{1}{\beta }\right)\frac{\partial^{2}u}{\partial y^{2}}-\frac{\sigma B_{0}^{2}}{\rho_{f}}u-\frac{\mu_{f}}{\rho_{f}K}u+\frac{g}{\rho_{f}}\left[\rho_{f}\beta \left(T-T_{\infty }\right)-\left(\rho_{p}-\rho_{f}\right)\left(C-C_{\infty }\right)\right],$$ (2)

$$u\frac{\partial T}{\partial x}+v\frac{\partial T}{\partial y}=\alpha_{f}\left(1+\frac{16\sigma^{*}T_{\infty }^{3}}{3kk^{*}}\right)\frac{\partial^{2}T}{\partial y^{2}}+\tau D_{B}\left(\frac{\partial T}{\partial y}\frac{\partial C}{\partial y}\right)+\frac{\tau D_{T}}{T_{\infty }}{\left(\frac{\partial T}{\partial y}\right)}^{2}+\frac{\alpha_{f}\mu_{f}}{k}{\left(\frac{\partial u}{\partial y}\right)}^{2}+\left(\frac{\alpha_{f}\sigma B_{0}^{2}}{k}+\frac{\alpha_{f}\mu_{f}}{kK}\right)u^{2},$$ (3)

$$u\frac{\partial C}{\partial x}+v\frac{\partial C}{\partial y}+kr\left(C-C_{\infty }\right)=D_{B}\frac{\partial^{2}C}{\partial y^{2}}+\frac{D_{T}}{T_{\infty }}\frac{\partial^{2}T}{\partial y^{2}},$$ (4)

with boundary conditions:

$$\left.\begin{array}{l} u=ax,\;\;\;v=0,\;\;\;T=T_{w},\;\;\;C=C_{w}\;\;\;at\;\;\;y=0\\ u\to 0,\;\;\;T\to T_{\infty },\;\;\;C\to C_{\infty }\;\;\;as\;\;\;\;\;\;y\to \infty \end{array}\right\},$$ (5)

Here, the velocity components are *u* and *v* along *x*– and *y*–directions respectively, *v*_*f*_ is the kinematic viscosity, *ρ*_*p*_ is the density of the nanoparticle, *ρ*_*f*_ is the density of the fluid, *D*_*B*_ is coefficient of Brownian diffusion, *D*_*T*_ is the coefficient of thermophoresis diffusion, *α*_*f*_ is the thermal diffusivity, *kr* is the chemical reaction term, *C* is the concentration, *T* is the temperature, *C*_*w*_ is the surface concentration and *μ*_*f*_ is the fluid viscosity.

The correspondence transformations are defined as [34]:

$$\psi \left(x,y\right)=\alpha_{f}Ra_{x}^{1/4}f\left(\xi \right),\;\;u=\frac{\partial \psi }{\partial y},\;\;v=-\frac{\partial \psi }{\partial x},\;\;\theta \left(\xi \right)=\frac{T-T_{\infty }}{T_{w}-T_{\infty }},\;\;\phi \left(\xi \right)=\frac{C-C_{\infty }}{C_{w}-C_{\infty }},\;\;\xi =\frac{y}{x}Ra_{x}^{1/4}.$$ (6)

Using the above resemblance substitution, the leading equations are reduced as:

$$\left(1+\frac{1}{\beta }\right)f^{\prime \prime \prime }+\frac{1}{4\text{Pr}}\left(-2{f^{\prime }}^{2}+3ff^{\prime \prime }\right)-\left(M+\frac{1}{Da}\right)f^{\prime }-Nr\phi +\theta =0,$$ (7)

$$\left(1+\frac{4}{3}Rd\right)\theta^{\prime \prime }+\frac{3}{4}f\theta^{\prime }+Ec\;\text{Pr}\left({f^{\prime \prime }}^{2}+\left(M+\frac{1}{Da}\right){f^{\prime }}^{2}\right)+Nb\theta^{\prime }\phi^{\prime }+Nt{\theta^{\prime }}^{2}=0,$$ (8)

$$\phi^{\prime \prime }+\frac{Nt}{Nb}\theta^{\prime \prime }+\frac{3}{4}Lef\phi^{\prime }-LeK\phi =0,$$ (9)

with

$$f\left(0\right)=0,\;\;f^{\prime }\left(0\right)=\lambda ,\;\;\phi \left(0\right)=1,\;\;\;\theta \left(0\right)=1,\;\;f^{\prime }\left(\infty \right)=0,\;\;\phi \left(\infty \right)=0,\;\;\;\theta \left(\infty \right)=0.$$ (10)

Here, the magnetic parameter is indicated by $M\left(=\frac{\sigma_{f}B_{0}^{2}x^{2}}{\mu_{f}Ra_{x}^{1/2}}\right)$, Eckert number is denoted by $Ec\left(=\frac{u_{w}^{2}}{C_{pf}\left(T_{w}-T_{\infty }\right)}\right)$, thermal radiation is represented by $Rd\left(=\frac{4\sigma^{*}T_{\infty }^{3}}{kk^{*}}\right)$, Prandtl number is signified by $\text{Pr}\left(=\frac{\nu_{f}}{\alpha_{f}}\right)$, Brownian motion is indicated by $Nb\left(=\frac{\tau D_{B}\left(C_{w}-C_{\infty }\right)}{\alpha_{f}}\right)$, $Nr\left(=\frac{\left(\rho_{p}-\rho_{f}\right)\left(C_{w}-C_{\infty }\right)}{\rho_{f}\beta \left(T_{w}-T_{\infty }\right)}\right)$ is the buoyancy ratio parameter, thermophoresis term is denoted by $Nt\left(=\frac{\tau D_{T}\left(T_{w}-T_{\infty }\right)}{\alpha_{f}T_{\infty }}\right)$, the stretching/shrinking parameter is indicated by $\lambda \left(=\frac{ax^{2}}{Ra_{x}^{1/2}\alpha_{f}}\right)$, Lewis number is denoted by $Le\left(=\frac{\alpha_{f}}{D_{B}}\right)$, and the porous medium parameter is represented by $Da\left(=\frac{KRa_{x}^{1/2}}{x^{2}}\right)$.

The mathematical expressions for the physical quantities are stated as:

$$C_{f}=\frac{\tau_{w}}{\mu_{f}\alpha_{f}Ra_{x}^{3/4}},\;\;Nu=\frac{1}{k}\frac{xq_{w}}{\left(T_{w}-T_{\infty }\right)},\;\;Sh=\frac{1}{D_{B}}\frac{xq_{m}}{\left(C_{w}-C_{\infty }\right)},$$ (11)

where *τ*_*w*_, *q*_*w*_ and *q*_*m*_ are rebound as:

$$\tau_{w}=\left(1+\frac{1}{\beta }\right){\left.\frac{\partial u}{\partial y}\right|}_{y=0},\;\;q_{w}={\left.-\left(k+\frac{16\sigma^{*}T_{\infty }^{3}}{3k^{*}}\right)\frac{\partial T}{\partial y}\right|}_{y=0},\;\;q_{m}=-D_{B}{\left.\frac{\partial C}{\partial y}\right|}_{y=0}.$$ (12)

Eq (11) is reduced as:

$$C_{f}Ra_{x}^{1/4}=\left(1+\frac{1}{\beta }\right)f^{\prime \prime }\left(0\right),\;\;\frac{Nu}{Ra_{x}^{1/4}}=-\left(1+\frac{4}{3}Rd\right)\theta^{\prime }\left(0\right),\;\;\frac{Sh}{Ra_{x}^{1/4}}=-\phi^{\prime }\left(0\right),$$ (13)

where $Ra_{x}=\frac{\left(1-\varphi \right)g\beta \left(T_{w}-T_{\infty }\right)x^{3}}{\nu_{f}\alpha_{f}}$ is the local Rayleigh number.

## 3. HAM solution

The initial guesses and linear operators are taken as:

$$f_{0}\left(\xi \right)=\lambda \left(1-e^{-\xi }\right),\;\;\theta_{0}\left(\xi \right)=e^{-\xi },\;\;\phi_{0}\left(\xi \right)=e^{-\xi }.$$ (14)

$$L_{f}=f^{\prime \prime \prime }-f^{\prime },\;\;L_{\theta }=\theta^{\prime \prime }-\theta ,\;\;L_{\phi }=\phi^{\prime \prime }-\phi .$$ (15)

with properties:

$$L_{f}\left(q_{1}+q_{2}e^{\xi }+q_{3}e^{-\xi }\right)=0,\;\;L_{\theta }\left(q_{4}e^{\xi }+q_{5}e^{-\xi }\right),\;\;L_{\phi }\left(q_{6}e^{\xi }+q_{7}e^{-\xi }\right)=0,$$ (16)

where *q*_1_ − *q*_7_ are the constants of general solution.

## 4. HAM convergence

**Fig 2:**
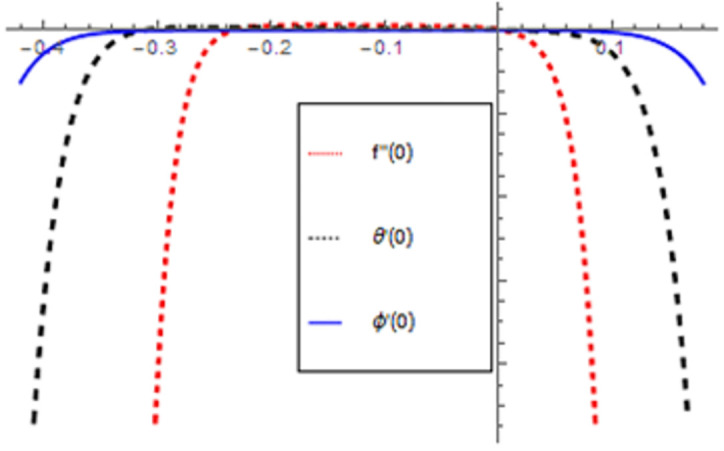
ℏ–curves for *f*′(*ξ*), *θ*(*ξ*) and *ϕ*(*ξ*).

HAM provides the convergence of the series solutions. The auxiliary factor ℏ performs an important character in adjusting and controlling the region of convergence of our series solutions. Thus, we have schemed the ℏ–curves for *f*′(*ξ*), *θ*(*ξ*) and *ϕ*(*ξ*) in Fig 2. The region of convergence for *f*′(*ξ*), *θ*(*ξ*) and *ϕ*(*ξ*) are 0.25 ≤ ℏ_*f*_ ≤ 0.0, −0.32 ≤ ℏ_*θ*_ ≤ 0.05 and −0.34 ≤ ℏ_*ϕ*_ ≤ 0.1 respectively.

## 5. Discussion of results

This work describes the improvement of energy and mass transmission for a mixed convection Casson fluid flow upon an extending sheet using an absorbent medium. The fluid flow has exposed the impressions of thermophoresis and Brownian motion by using the idea of Buongiorno’s model. The influence of chemical reaction has also been incorporated mathematically in the concentration equation. The equations that regulated the flow problem have transformed to dimensionless notation with suitable similarity variables. The solution has been determined by HAM. The codes for HAM are incorporated in MATHEMATICA 12.0 software. The authentication of the present analysis is represented by Table 1. The effect of various substantial constraints on the fluid flow profiles has been reviewed in the subsequent lines. To support our discussion, a graphical view of different profiles has also been presented.

**Table 1:** Validation of the skin friction values with previously published results when *M* = 1.0, *Ec* = 0.1, *Da* → ∞, and *β* → ∞.

*Nr*	*λ*	*f*″(0)	
		Makinde et al. [34]	Presents results
0.0	-0.5	2.35476	2.354765
0.3		2.21267	2.212673
0.5		2.11589	2.115891
0.0	0.0	1.73117	1.731173
0.3		1.60462	1.604624
0.5		1.51848	1.518482
0.0	0.5	1.03430	1.034305
0.3		0.92053	0.920532
0.5		0.84380	0.843801

**Fig 3:**
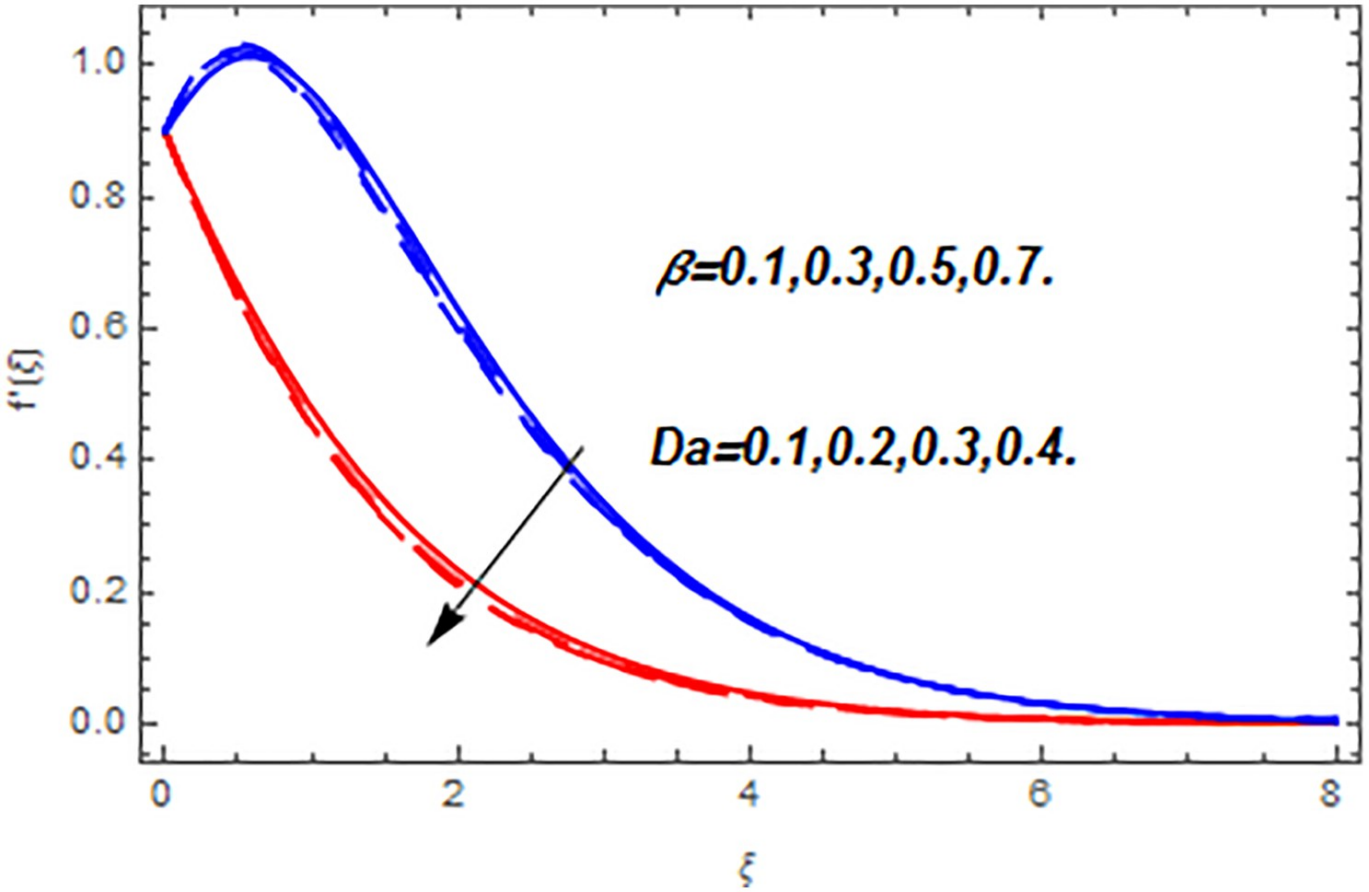
Flow profiles versus Darcy number and Casson parameter.

**Fig 4:**
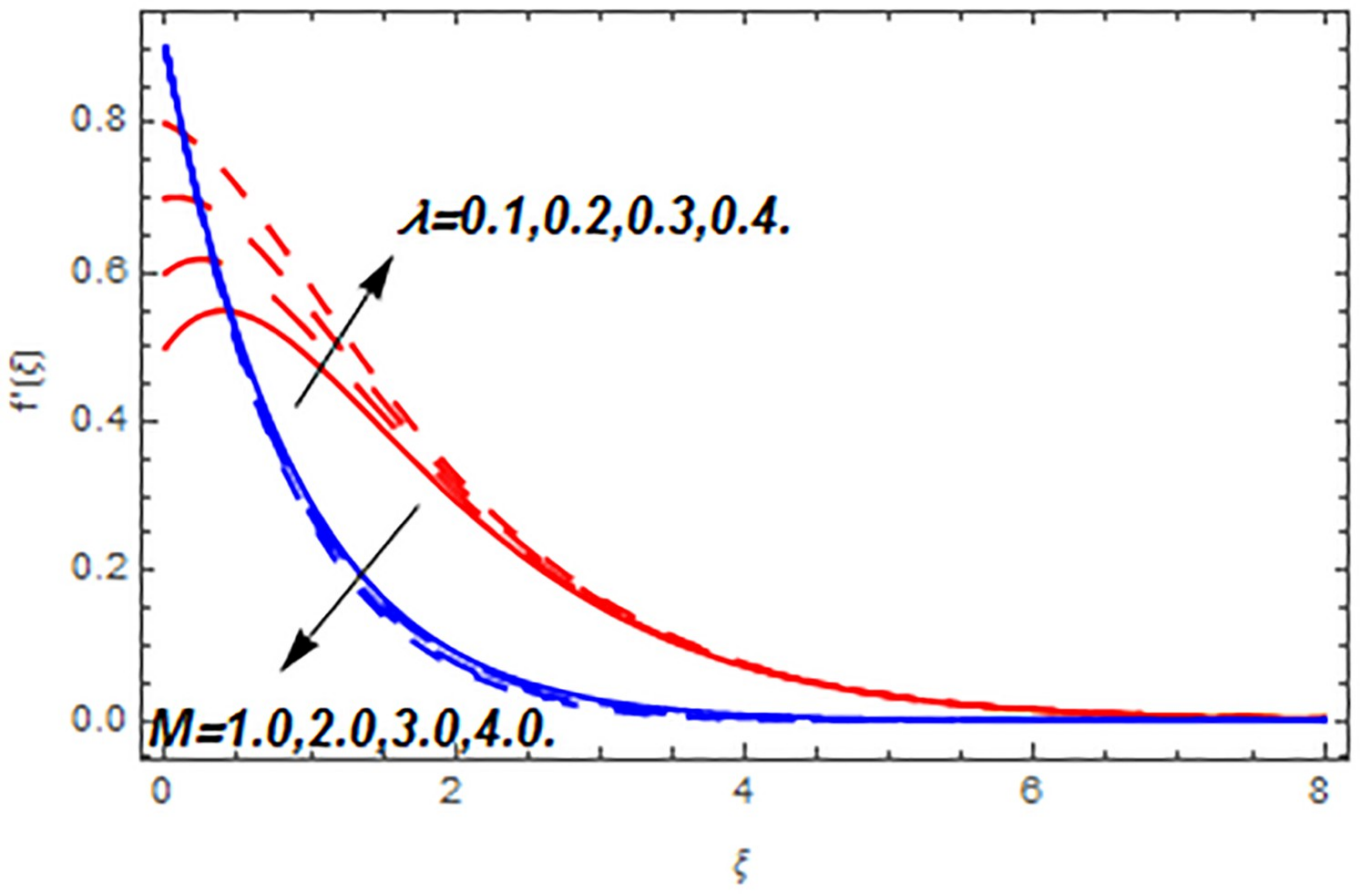
Flow profiles versus magnetic and stretching parameters.

**Fig 5:**
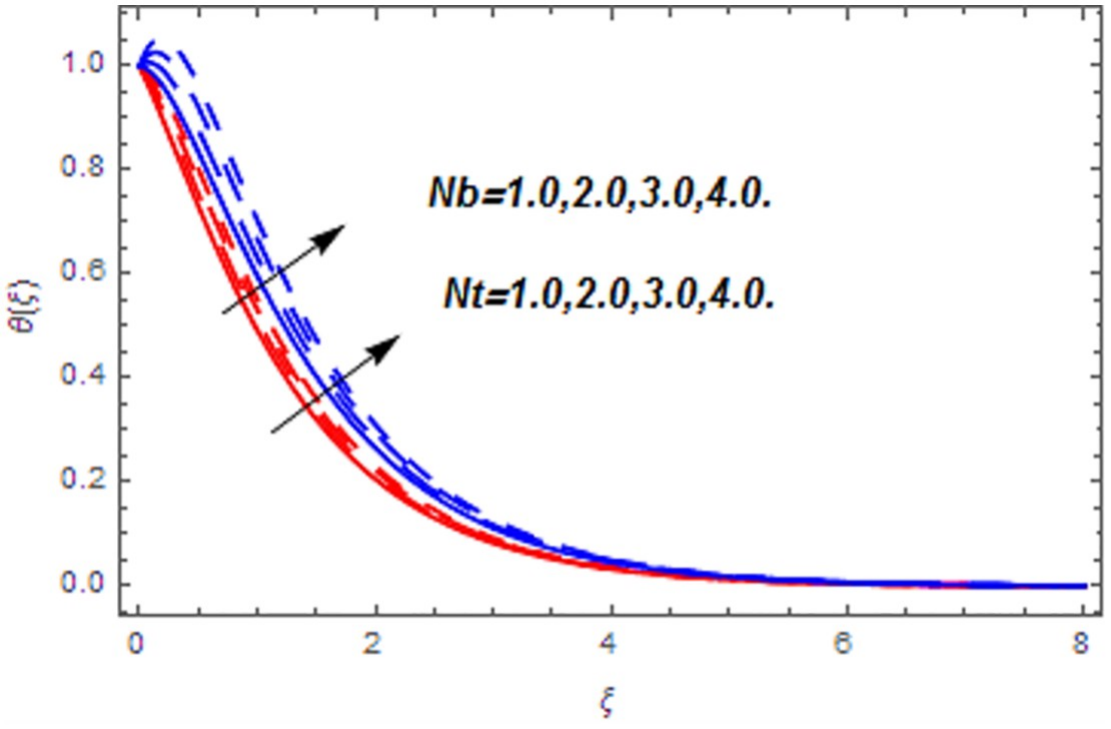
Thermal profiles versus Brownian motion and thermophoresis parameters.

**Fig 6:**
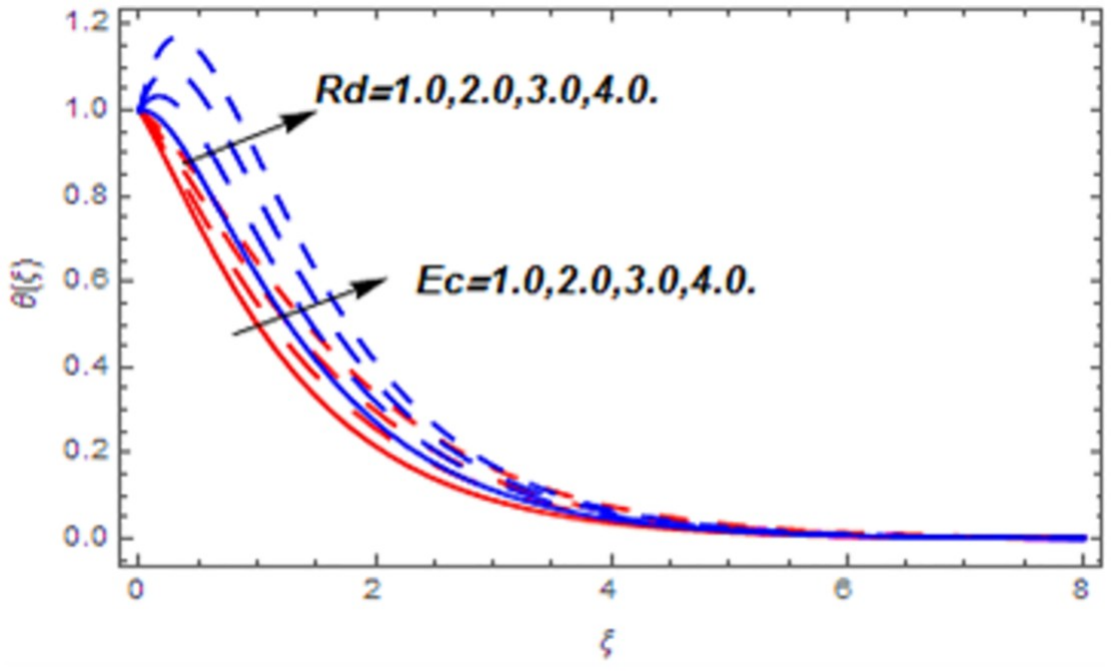
Thermal profiles versus Eckert number and radiation parameter.

**Fig 7:**
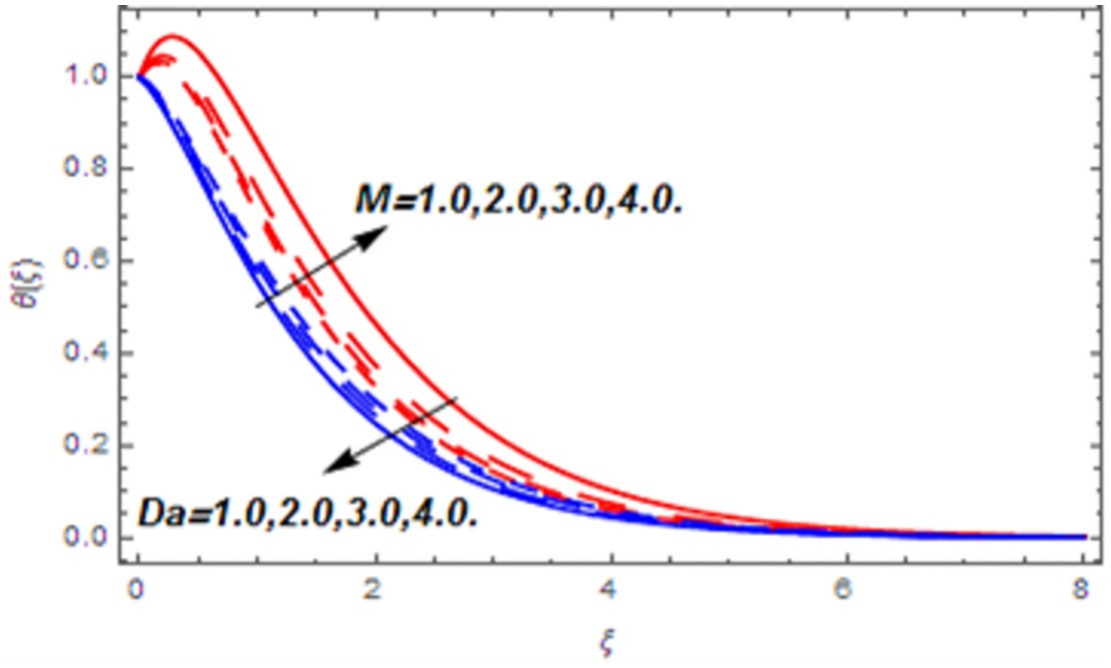
Thermal profiles versus Darcy number and magnetic parameter.

**Fig 8:**
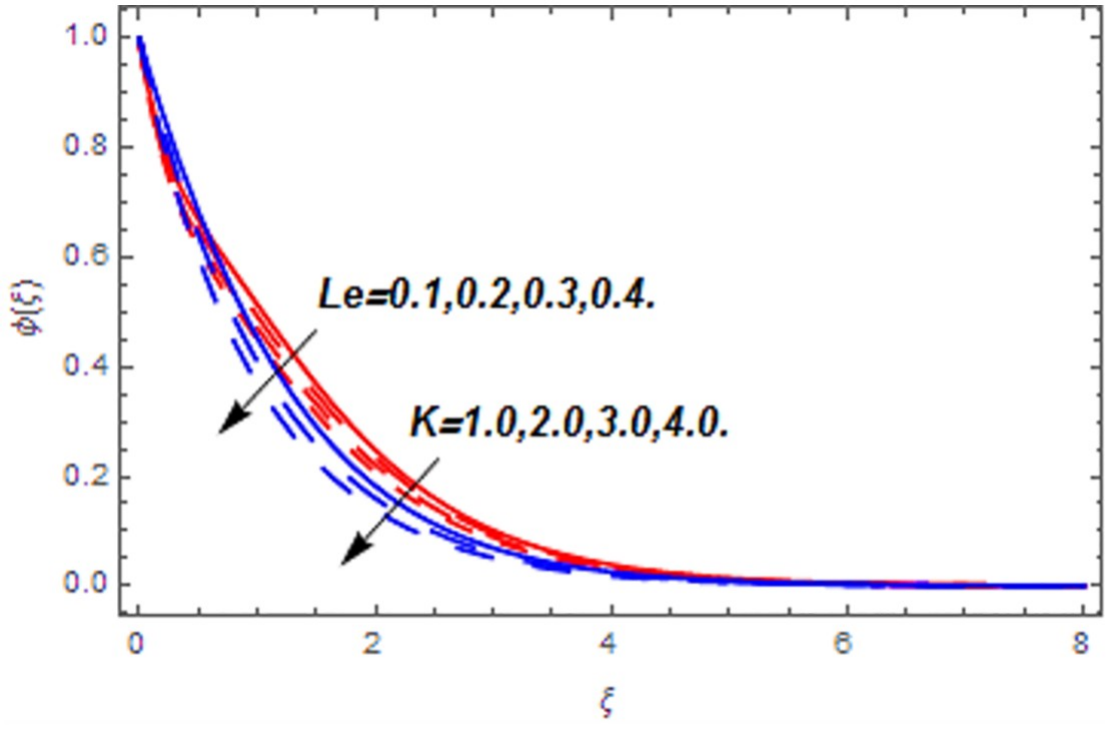
Concentration profiles versus Lewis number and chemical reaction parameter.

**Fig 9:**
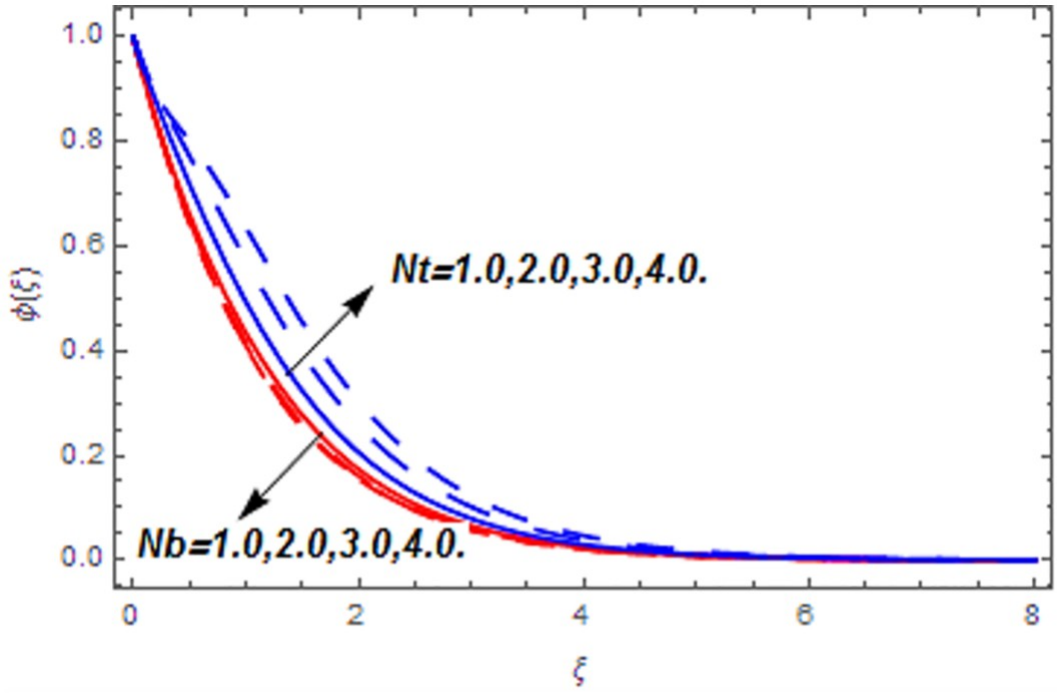
Concentration profiles versus Brownian motion and thermophoresis parameters.

**Fig 10:**
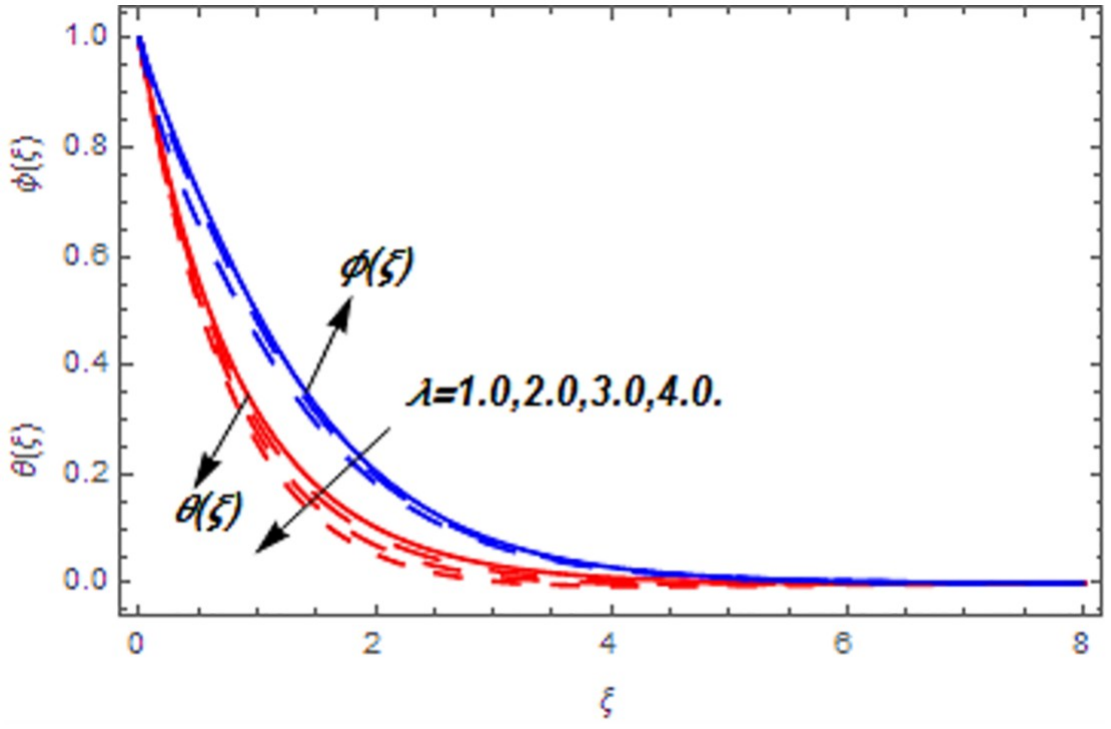
Impact of stretching parameter on thermal and concentration profiles.

**Fig 11:**
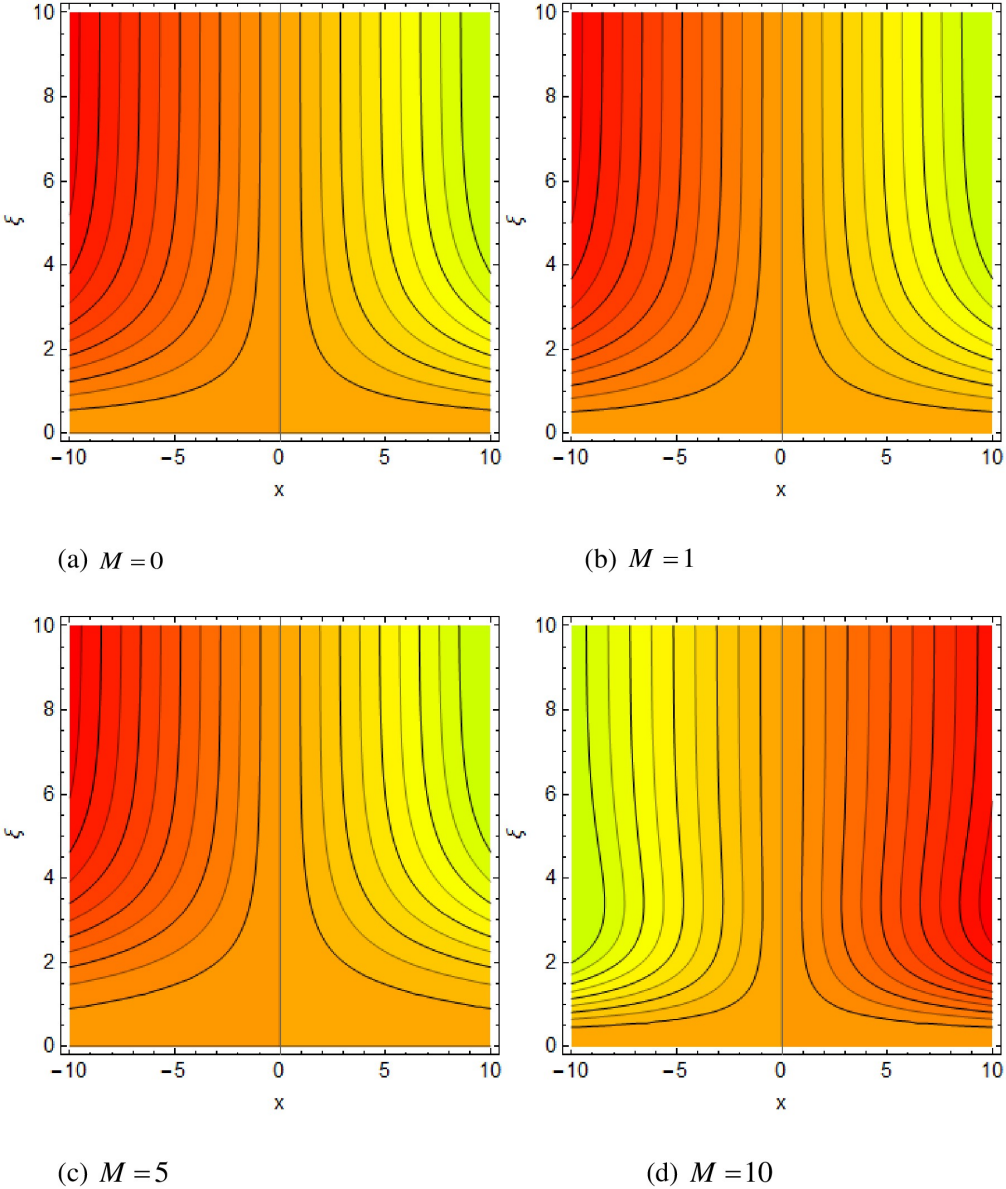
(a–d) Impact of magnetic field on streamlines when Pr = 5.0, *λ* = 0.5, *Da* = 1.0, *Ec* = 0.1, *Nb* = 0.2, *Nt* = 0.2, *Le* = 0.3, *Nr* = 0.2, *Rd* = 1.3 and *Ra* = 10^3^.

Fig 3 portrays the influence of Darcy number *Da* and Casson parameter *β* upon the velocity profiles. It has been spotted from this figure that with enlargement in Darcy number the void spaces in the surface will increase that offers more resistance to the fluid motion. In this physical phenomenon, the velocity characteristics decline. On the other hand, the declining impact of Casson coefficient *β* on the velocity field is depicted. The reason behind the declining impact on velocity profile is that the augmenting Casson parameter augments the viscosity of the fluid flow which yields the reduction in velocity of the fluid flow. Also, the increasing Casson parameter (say *β* → ∞) leads to the viscous fluid. Fig 4 reveals the influence of magnetic *M* and stretching constraint *λ* upon the velocity field. Since an augmentation in the values of *M* creates the Lorentz force which produces the opposing force to the fluid flow. This opposing force reduces the velocity of the fluid flow. Therefore, a declining effect of magnetic term is depicted. On the other hand the growth in *λ* leads to an augmentation in the flow of fluid. It is obvious that the increasing stretching parameter augments the stretching phenomenon of the proposed geometry which consequently augments the velocity of the fluid. Thus an augmenting impact is observed here. Fig 5 presents the upshot of Brownian motion and thermophoresis upon energy profile. An augmentation in *Nb* grows up the thermal flow of fluid. Actually, it can be explained as a growth in *Nb* augments the chaotic motion amongst the particles, due to which the kinetic energy of the particles transform to heat energy. In this process the thermal profile of the particles grow up. Similarly, a growth in thermophoresis constant *Nt* supports the energy field. Actually for upsurge values of *Nt* the difference in temperature of the fluid grows up due to which more heat will transfer from hotter to colder region. Hence augmentation in *Nt* increases the thermal profile for fluid flow system. Fig 6 depicts the behavior of thermal profile in response of Eckert number *Ec* and radiation *Rd*. For augmenting values of *Ec* the transport energy of fluid flow system grows up that causes the alteration of kinetic energy to heat energy. In this physical phenomenon the thermal profiles upsurge. Similarly growth in radiation parameter pushes the temperature of the fluid in forward direction. Actually, growth in *Rd* causes a maximum release of heat energy to flow direction that gives strength to the thickness of thermal layer, and ultimately grows up the thermal flow profile. Fig 7 portrays the influence of Darcy number *Da* and magnetic parameter *M* upon thermal profiles. The higher values of *Da* causes a higher permeability in porous surface that leads to a drop down in thermal conduction. In this physical phenomenon the thermal profiles decline. Fig 7 also depicts an upsurge in thermal profile due to growth in magnetic field. Actually, the extension in magnetic parameter leads to generation of Lorentz force that causes a reduction in the flow profile due to resistive force. In this process a maximum heat flow takes place and causes an augmentation in thermal profile. Fig 8 expresses the upshot of Lewis number *Le* and chemical reaction factor *K* upon concentration profiles. The augmenting values of *Le* and *K* causes a reduction in the diffusivity of mass of fluid, due to which less transmission of mass takes place that weakens the concentration boundary layer thickness. On the other hand, the molecular diffusivity also suppresses with the growing in chemical reaction term which eventually decays the concentration of the fluid flow. Therefore, a declining impact is depicted here. Fig 9 examines the impacts of Brownian motion *Nb* and thermophoresis factor *Nt* upon the concentration profiles. The higher values of *Nb* has a reverse impact on the mass transfer. Actually, with the augmentation in *Nb* the random motion boosts due to which fluid particles are colliding more frequently. In this phenomenon more resistance is experienced by the fluid motion that weakens the concentration boundary layer thickness that ultimately drops the mass profile as portrayed in Fig 9. The concentration gradient within the fluid particles maximizes for higher values of *Nt*, due to which more mass transfers from higher to lower concentration region. In this physical process concentration profiles grow up. Fig 10 demonstrates the influence of stretching parameter *λ* upon the energy and mass profiles. Form this figure it is observed that the higher values of *λ* has an adverse effect upon both profiles. Actually, the increasing stretching parameter decrease the thermal and mass boundary layer thicknesses and thus the thermal and concentration profiles decline. Fig 11a–11d displays the impact of magnetic field on streamlines. Fig 11a shows the streamlines of the Casson fluid for the case of non-magnetized flow. Here, the streamlines are close to each other which show the heightening impact in velocity of the Casson fluid flow. Fig 11b–11d shows the streamlines of the Casson fluid for the case of magnetized flow. Here, for the increasing magnetic field, the streamlines becomes apart from each other which shows the reducing impact in velocity of the Casson fluid flow. Actually, the greater magnetic field produces resistive force to the fluid flow which reduces the velocity profile of the fluid flow. The surface drag force, heat, and mass transfer rates are calculated through a numerical approach and are displayed in Table 2. The resistive forces augmented with the rising values of *β*, *M*, and *Da*, while the drag force fall due to the higher values of *Rd* and *Ec*. In fact, the viscous forces are not dominant in the presence of the accumulative values of these parameters. Similarly, the heat transmission rate reduce due the larger amount of *β* and *Da*, while show dominancy in the case of the larger values of *Rd*, *Ec*, *M*, *Nt* and *Nb*. The mass transport rate decline with the increasing values of *Le* and *Nt*, while rises with the greater values of *Nb*.

**Table 2:** Statistical assessments of skin friction, energy transition and mass transmission rates.

*β*	*Da*	*M*	*Rd*	*Ec*	*Nb*	*Nt*	*Le*	$\left(1+\frac{1}{\beta }\right)f^{\prime \prime }\left(0\right)$	$-\left(1+\frac{4}{3}Rd\right)\theta^{\prime }\left(0\right)$	−*ϕ′(0)*
0.1	0.1	0.1	0.1	0.1	0.1	0.1	0.1	0.83827	1.73421	1.68537
0.2								0.97432	1.70053	1.68537
0.3								1.09264	1.62473	1.68537
	0.2							0.97630	1.72243	1.68537
	0.3							1.187614	1.71731	1.68537
		0.2						1.034123	1.74213	1.68537
		0.3						1.376413	1.75102	1.68537
			0.2					0.83716	1.842301	1.68537
			0.3					0.83802	1.95042	1.68537
				0.2				0.837231	1.78337	1.68537
				0.3				0.836012	1.83341	1.68537
					0.2			0.83827	1.75431	1.77143
					0.3			0.83827	1.77523	1.89331
						0.2		0.83827	1.78783	1.65431
						0.3		0.83827	1.83910	1.62402
							0.2	0.83827	1.73421	1.55268
							0.3	0.83827	1.73421	1.42423

## 6. Conclusion

This work describes the improvement of energy and mass transmission for a mixed convection Casson fluid flow upon a stretching porous sheet. The fluid flow is considered with the impacts of thermophoresis and Brownian motion by using the idea of Buongiorno’s model. The influence of chemical reactions has also been incorporated in the concentration equation. The equations that regulate the flow problem have been transmuted to dimensionless forms by using suitable similarity variables. The solution has been determined by HAM. The impact of numerous substantial constraints upon different profiles of the flow systems has been discussed in detail. The key points of the proposed analysis are listed as:

- The augmented Darcy number, Casson and magnetic parameters have declined the velocity of the Casson fluid flow.
- Growth in Brownian motion augments the chaotic motion amongst the particles, due to which the kinetic energy of particles transforms to heat energy which consequently augmented the thermal profile, while reduced the concentration profile.
- Augmentation in thermophoresis parameter has increased the temperature and concentration profiles.
- The augmented radiation parameter, magnetic parameter, and Eckert number have augmented the thermal profile, while the higher Darcy number has declined the thermal profile.
- The growing values of Lewis number and chemical reaction parameter cause a reduction in the diffusivity of mass of fluid, due to which less transfer of mass takes place that weakens the concentration layer thickness and declines the concentration profiles.
- The higher stretching parameter has declined the thermal and concentration profiles.
